# Ethics and Health Security in the Australian COVID-19 Context: A Critical Interpretive Literature Review

**DOI:** 10.1007/s11673-023-10255-6

**Published:** 2023-11-08

**Authors:** Anson Fehross, Kari Pahlman, Diego S. Silva

**Affiliations:** Sydney Health Ethics, University of Sydney, Edward Ford Building, A27 Fisher Rd, Sydney, NSW 2006 Australia

**Keywords:** Security, Harm, Safety, Transparency, Governance, Pandemic

## Abstract

*Background* The concept of “health security” is often used to motivate public health responses, yet the ethical values that underpin this concept remain largely unexamined. The recent Australian responses to COVID-19 serve as an important case study by which we can analyse the pre-existing literature to see what ethical values shaped, and continue to shape, Australia’s response. *Methods* We conducted a critical interpretive literature review of academic and grey literatures within key databases, resulting in 2,220 sources. After screening for duplicates and relevance, we analysed ninety-six sources. *Results* First, risk and uncertainty are a leading focus, with a heavy concentration on risks to life and health. Second, free movement, safety, and security were recurringly emphasized, albeit narrowly focused upon the safety of the population. Third, legitimacy was a recurring theme, and it is here that discussions of “health security” figured highly. *Conclusion* Discussions of harm from government and associated official bodies fail to adequately distinguish between various senses of harm. Moreover, while the literature often discusses the balancing of rights, the steps involved in the weighing of these rights is rarely adequately explained and defended. We suggest that decision-makers should endeavour to clearly identify and defend the values undergirding their decisions in the public sphere.

As evidenced by the actions of health bodies and government taken in response to the recent COVID-19 pandemic, Australia’s approach to health security centres upon strategies of containment, including strict control of travel. As an island nation, Australia’s natural borders protect the populace more effectively than countries that share land borders (Bashford 2003; Bennett 2009; Babones 2020a; Blakely et al. 2020; Boyd, Baker, and Wilson 2020; House of Representatives a; Commonwealth of Australia 2020; Legislative Assembly of the Northern Territory 2020; Rang et al. 2020; Swan 2020; Fotheringham et al. 2021; Ng and Gray 2021; Department of the Prime Minister and Cabinet 2006; Holley, Coatsworth, and Lipman 2021). Appeals to “health security” are used—in Australia and many other countries and regions—as an impetus for these kinds of restrictive public health actions.

The concept of health security is primarily invoked to justify state actions to ensure the safety of the populace (Aldis 2008; Davis, Stephenson, and Flowers 2011; Rushton 2011; Stoeva 2020). As Davis et al. note, however, there is little agreement regarding its meaning: “For some it means bioterrorism, but for others it seems to mean almost everything pertaining to public health” (Davis, Stephenson, and Flowers 2011, 916). Moreover, this concept is underpinned by an array of implicit and unexamined ethical values which give rise to contestable decisions. We here understand values to refer to those organizing beliefs or principles that motivate and justify the reasoning process that lay beneath some action (Weber 1978). Of course, the nature of values is often ambiguous or contested, and we choose to narrow our focus to primarily *ethical* values—that is the normative evaluation of actions as contributing to, or expressive of, a particular view of the good or right. Actions are thus reflective of and express values even when the values in question are neither fully articulated nor defended. Yet there has been little attention paid in the literature to considering the values underlying the concept of health security (Pahlman et al. 2022). Given health security’s highly contentious nature, understanding the values that potentially animate actions under its guise will help not only clarify the concept of health security but also help justify (or not) those measures that are enacted in its name.

Australia’s COVID-19 response provides a rich opportunity to evaluate the ethical values of health security as it relates to pandemics. There are at least two recent salient examples where discussions of health security served to motivate Australia’s response. First, on January 29, 2020, Australia’s then Prime Minister Scott Morrison announced that Australian citizens and permanent residents within China’s Hubei Province would be voluntarily evacuated to Australia’s Christmas Island Immigration Detention Centre—famous for its use, until recently, to sequester asylum seekers away from the Australian mainland (Shelton and Birtles 2020; Roy and Doherty 2020; Bennett 2021). Second, in May 2021, the Federal Government enacted temporary emergency laws threatening fines and imprisonment and refusing re-entry to Australians in India as a response to the Delta variant (Maclean 2021). As we will show, such decisions need a clear ethical justification since they directly impact the lives of Australian citizens and residents and interfere with their implied right to freedom of movement—more specifically, the freedom to return to one’s country of residence. This paper aims to provide an analysis of how discussions utilizing “health security” and related concepts potentially shaped and motivated Australia’s response to COVID-19. It also aims to clarify the underlying values that guide these discussions of health security and its applications in practice. The first step towards any ethically robust response must be to do this kind of conceptual clarification. To achieve these goals, we first identify and describe, via the employment of a critical interpretive review, those unexamined ethical values that underpinned Australia’s response to the growing threat of COVID-19. We identify a variety of value judgements within three broad themes: first, the nature of risk and uncertainty in the face of a pandemic; second, the weighing of safety and security against the value of free movement; third, how public health measures are legitimated. Finally, we offer an ethically informed critique of the ways these values have been used to motivate and justify these responses. To this end, we collect our critiques under three areas worthy of scrutiny. First, we explore how the literature has implicitly adopted a narrow conception of the nature of harm, neglecting alternative renderings that have an equal claim to prominence. Second, we demonstrate that safety and security are granted more weight than alternate goods, often without justification or argument. Third, we examine how discussions of balancing the rights of individuals against the broader community are often superficial. Ultimately, our goal is to provide an analysis of literature that predated and likely shaped the COVID-19 response within Australia, alongside an analysis of how dominant values expressed within these literatures were put into practice explicitly and implicitly. We are presuming that the values that underpinned health security and pandemic planning prior to the onset of COVID-19 had some role in shaping Australia’s actual response vis-à-vis its borders and its perceived regional and global responsibilities. Detailed, and explicitly ethical, attention must be paid to these responses to avoid the uncritical enaction of decisions that can, and did, result in significant harms to the populace. To answer the question of whether these harms are justified we must, first, uncover the rationale that laid beneath the decisions that gave rise to these harms. Our approach here represents an attempt to uncover what values could have motivated such decisions and the extent to which they were articulated clearly.

## Methods

This paper utilizes Dixon-Woods’ critical interpretive literature review methodology (i.e., critical interpretive synthesis, hereafter “CIS”) as adapted by McDougall for bioethics research (Dixon-Woods et al. 2006; McDougall 2015). Literature reviews of this sort are “thoughtfully-designed and thorough, but not systematic in the sense of aiming to assemble every article relevant to the research question” (McDougall 2015, 525). The goal is not to simply collate and count instances of arguments or ideas but rather to describe key insights and reflections about a field of literature, thereby applying critical theory and arguments to augment a subject area’s core body of knowledge. A CIS methodology thus aims to avoid a narrowly quantitative methodology, instead seeking to bring together qualitative and quantitative analysis of a body of literature, providing a synthesized interpretation of the available literature. A CIS analysis allows the key claims to rise out of the evidence, as well as licensing explicit *argument* regarding key themes, drawing out concepts, and developing an overall account. As a result, a CIS methodology offers a real benefit when addressing material that spans multiple disciplines that approach the same topics, with an eye to tying recurring threads into an overall narrative. This process is explicitly evaluative, as the process aims to critique the literature as a whole rather than providing individuated criticism.

We follow McDougall in utilizing a modification of CIS—a methodology she dubs a critical interpretive *review* (McDougall 2015). As summarized by McDougall, there are key similarities in methodology between a CIS and a critical interpretive review, as each approach:

1. Answers a specific research question, which may have been refined and determined during the literature review process, and
2. Analyses the literature as a whole as well as analysing individual findings and arguments within that literature, and
3. Does not utilize rigid quality assessment criteria but comments within the review itself on quality issues, and
4. Generates theory and puts forward an argument about the literature, and
5. Captures all of the key ideas in the existing literature that are relevant to the research question and records and reports the search strategy. (McDougall 2015, 527)

The goal of this type of analysis is to generate genuine insights regarding the literature as a whole. This involves picking out the “key ideas”—“ideas that are influential in the discussion to date and/or uniquely insightful in relation to the research question”—that run through the relevant literature (McDougall 2015, 527). This distinguishes the critical interpretive review methodology from a *systematic* review, where *every* recurring viewpoint is intended to be captured.

Invariably, determining what ideas are “key” will involve subjective judgement and risks the charge of bias. As McDougall notes,

undertaking a critical interpretative review as I have described it includes activities such as deciding which parts of the existing literature qualify as key ideas, and assessing the quality of the existing literature. The results of such activities may differ between individuals, depending on their own working definitions of concepts such as “key ideas” … (McDougall 2015, 527)

To this end, we adopted the working definition that a key idea will be determined by how highly it figures in a number of contexts, and to what extent other authors—especially from the academic literature—take the concept to undergird and motivate governmental and health responses to pandemics. Despite this possible drawback, the critical interpretive review has proven uniquely useful in facilitating robust reviews of the literature with an eye to normative critique, as demonstrated by several recent studies (Essex 2016; Robson et al. 2019; Cascio, Weiss, and Racine 2020). As a result, we are confident that the methodology is the best available for our purposes.

We collected sources via ProQuest Central, SCOPUS, APO, Web of Science, PUBMED, EBSCOHost Medline, Proquest Social Services Abstracts, MAIS: Multicultural Australia and Immigration Studies via Informit Online, Embase (OVID SP), Global Health (OVID SP), Proquest Social Science Database, SSRN, EBSCO Sociology Source Ultimate, Global Health via OVID, INFORMIT (AGIS Plus Text), WESTLAW AU, and LEXIS ADVANCE Research (AU). Keywords were designed to capture sources discussing health security and quarantine practices in the Australian context. The first set of keywords chosen for the academic literature was narrow, involving permutations of the keywords: “health security” AND “public good” AND Australia AND quarantine. Our initial strategy was to consider how measures that appeal to health security would be understood as a public good, hence the addition of this keyword. As this narrow search string returned few results, we broadened our strategy. As our point of departure was the COVID-19 pandemic, the responses from various agencies within Australia, and the academic literature centring upon the Australian context, we settled on the following as our primary search string: (Australia OR “New South Wales” OR Victoria OR Queensland OR “Australian Capital Territory” OR “South Australia” OR “Northern Territory” OR “Western Australia” OR Tasmania), quarantine AND pandemic. We limited, where possible, our searches to abstracts that contained keywords, as attempted full-text searches produced too many results. We felt confident to do so, as we were deliberately seeking sources that dealt with pandemics and health security as a primary focus.

In searching the grey and academic literature, we engaged in an iterative process in establishing keyword strings to best capture the maximum number of relevant sources. Broader search strings, such as “health security” AND (quarantine OR pandemic), resulted in too many results to reasonably review (in this instance, 547 results were returned). As our focus in this study was upon pandemic restrictions in the context of health security, we narrowed our strings to return reasonably targeted results.

After returning the above results, we turned to the following strings to search for remaining literature missed in our initial search:

- Australia OR “Western Australia” OR “South Australia” OR Tasmania OR Queensland OR Victoria OR “New South Wales” OR “Australian Capital Territory” OR “Northern Territory”) AND pandemic
- COVID AND quarantine AND Australia
- Australia AND quarantine AND “health security”
- Australia AND pandemic
- Australia AND quarantine
- Australia AND pandemic AND quarantine

We chose a date range terminating in March 2022, with no defined beginning search date. Despite this project focusing upon responses to COVID-19, we wanted a sense of how health security was understood in Australia before the onset and early stages of the pandemic to better contextualize our analysis. Academic sources were included as these sources provide contextualization and rigour—rigour often lacking within purely governmental sources. Academic sources were usually focused on political theory, history, or law and were largely represented by research papers, literature reviews, empirical research, and commentaries on policy (especially from the legal context). We also sought out grey literature, limiting these searches to sources emanating from Australian government agencies, including interviews, doorstop announcements, and media releases, as well as reports from think tanks and other organizations. The reason we chose to include this variety of grey literature was because this is one of the primary ways that policy changes are introduced and defended by politicians within the Australian context. This methodology allowed for a detailed examination of political history, sociology, and policy surrounding health security and pandemic responses.

**Fig. 1:**
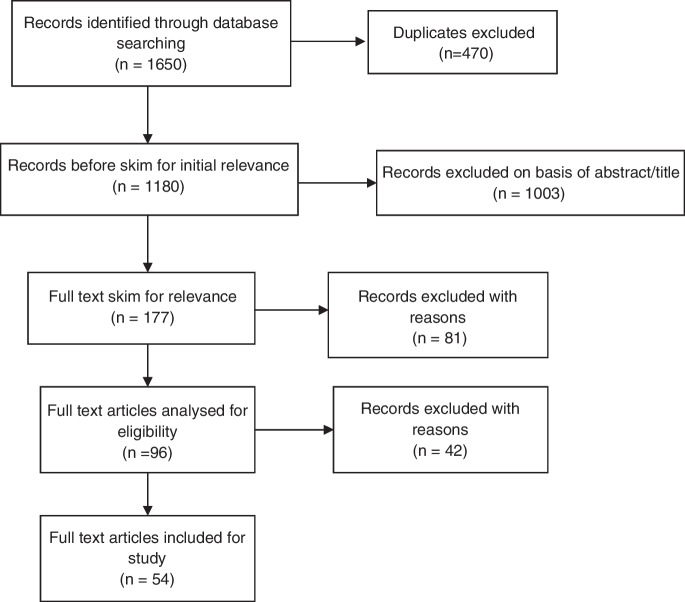
Academic literature

**Fig. 2:**
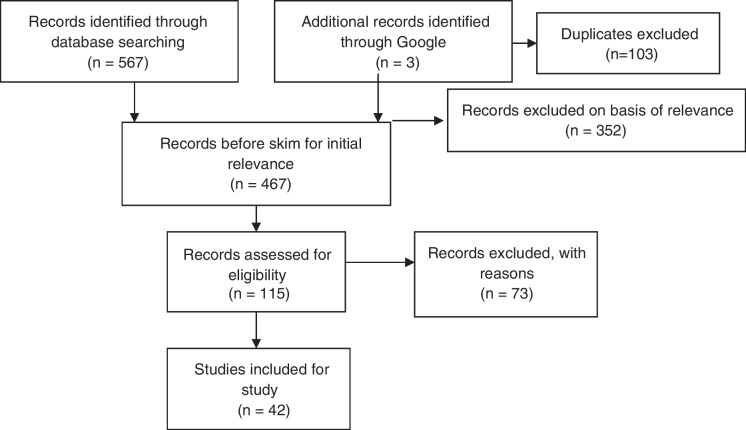
Grey literature

We initially identified 1650 academic papers, of which 470 were identified as duplicates and discarded. Next, we read titles and abstracts, excluding an additional 1003 articles that lacked focus on health security, pandemics, border control, and quarantine within the Australian context. As for grey literature, our searches returned 570 results, with 103 duplicates and we examined these for relevance by performing key word searches within the documents. Third, we skimmed and then read the remaining texts in full considering our overall goals, leaving a total of 96 sources. These processes are depicted in Figs. 1 and 2, below.

AF led the thematic analysis of the literature in conjunction with KP and DS. AF coded the literature, where the themes of each text were identified and summarized, gathering sources beneath three broad and recurring themes. These themes were verified by all authors, and source auditing was performed by KP and DS to ensure consistency.

## Results

We identified three key themes from this literature: risk and uncertainty; free movement, safety, and security; and legitimacy. Table 1, below, summarizes these key themes identified in this review with representative quotations. In what follows, our analysis will be critical, insofar as we make note of silences and decisions to highlight certain features ahead of others, with the overall goal of providing a summary of a variety of literature from a broad range of disciplines.

**Table 1:** Summary of themes with illustrative quotes

Theme	Illustrative quotes
Risk and uncertainty	“Action should be taken before there is evidence of sustained transmission of the novel coronavirus disease within the Australian community, it will be important to commence measures as quickly as possible, even though, due to the novel nature of the virus, it is unlikely that we will yet have a good understanding of the epidemiology, clinical severity and virology of the disease” (Department of Health 2020). “We argue that our public health agencies and governments must do better in transparently communicating the risks of COVID-19, the justifications for restrictive interventions, and the long-term all-things-considered goals of public health policy” (Jamrozik and Heriot 2020, 1169).
Free movement, safety, and security	“The [Parliamentary Joint Committee on Human Rights] considers that the measure, which is designed to prevent the spread of COVID-19, is likely to promote and protect the rights to life and health, noting that the right to life requires Australia to take positive measures to protect life and the right to health requires Australia to take steps to prevent, treat and control epidemic diseases. The committee notes that the measure may also limit the rights to freedom of movement and equality and non-discrimination. These rights may be subject to permissible limitations if they are shown to be reasonable, necessary and proportionate” (Parliamentary Joint Committee on Human Rights 2020b). “While the international human rights instruments, such as article 12 of the ICCPR, nowadays guarantee liberty of movement within a country, under international law it is possible for states to impose limitations to passage in order to safeguard public health” (Dzankic and Piccoli 2020).
Legitimacy	“Our first priority as a government is to protect the health and security of Australians. Alongside that, we also have a duty to protect and support those Australians who are overseas” (Hunt and Murphy 2020). “It is argued that encouraging voluntary compliance before restricting liberties would establish reasonable grounds for exercising discretion and imposing detention if it was eventually required. Voluntary compliance imposes reciprocal responsibilities on the community, such as the duty to obey public health orders[…]” (Pelkas 2010, 71).

### Risk and Uncertainty

A common value commitment expressed in the literature centred upon the government’s role to minimize the risk to the public of significant harm. Risk is a key part of pandemic planning, often articulated as a “vital systems security approach”—that is, dealing “with events whose probability cannot be precisely calculated, but whose consequences are potentially catastrophic” (Collier and Lakoff 2015, 22). In the context of pandemics, this approach focuses on identifying and mitigating risks to critical systems and infrastructure that are necessary for effective pandemic response and management, as well as ensuring the continuity of these systems. This kind of approach is central in the early stages of pandemics, when vital information such as the precise nature of the disease, its replication rate, and its precise effects is unknown (Blakely et al. 2020; Boyd, Baker, and Wilson 2020; Rang et al. 2020; Smith 2020; Buck 2020). As to how responses are organized, state and federal public health systems are tasked with setting up several risk management mechanisms, while the Federal Government is tasked with both organizing and taking decisive action at borders to prevent the spread of disease (Bashford 2003; Bennett 2009; Department of the Prime Minister and Cabinet 2006; Waller, Davis, and Stephenson 2016; Bennett, Carney, and Bailey 2012; Bashford and Howard 2004; Guy and Hocking 2006; Ly, Selgelid, and Kerridge 2007; Chen 2018; Brew and Burton 2004; Department of Health and Ageing 2006; Department of the Prime Minister and Cabinet 2019; Halton et al. 2021).

As to how these risks are understood, our investigation revealed a deep concentration on risks to health and life ahead of other considerations of harm (House of Representatives 2020b; Legislative Assembly of the Northern Territory 2020; Davis, Stephenson, and Flowers 2011; Department of Health and Ageing 2006; Department of the Prime Minister and Cabinet 2019; Senate Select Committee on COVID-19 2020a; Andrews 2021; Legislative Assembly of Western Australia 2020b ; Australian National Audit Office (ANAO) 2020; Joint Standing Committee on Foreign Affairs, Defence and Trade 2020; Kelly 2021; Hunt and Murphy 2020; Behm 2020; Department of the Prime Minister and Cabinet 2006; Australian House of Representatives 2008a; Morrison 2020; Hunt and Kelly 2020; Senate Select Committee on COVID-19 2021; Senate Select Committee on COVID-19 2020c; Bowen 2020; Morrison 2021; Babones 2020b; Department of Health 2020; Jamrozik and Heriot 2020; Chen 2018; Parliamentary Joint Committee on Human Rights 2020b; 2020a). Significant focus was also placed upon economic risks, including risks to international trade and domestic employment (Parliamentary Joint Committee on Human Rights 2020b; Ly, Selgelid, and Kerridge 2007; Brew and Burton 2004; Halton et al. 2021; Australian National Audit Office (ANAO) 2020; Joint Standing Committee on Foreign Affairs, Defence and Trade 2020; Behm 2020; Morrison 2021; Babones 2020b; Parliamentary Joint Committee on Human Rights 2020a; House of Representatives 2020a; House of Representatives 2021; Legislative Assembly of Western Australia 2020a; Letts 2006; Carney AO and Bennett 2013; Pelkas 2010; Boucher et al. 2021; Bennett 2021; Australian National Audit Office (ANAO) 2021; Stobart and Duckett 2021; Holley, Coatsworth, and Lipman 2021; Milne et al. 2021; Gussen 2021). This large concentration on only a narrow set of risks represents a value judgement on the part of government and health agencies. The fact that risks to economic systems, alongside those to health and life, were discussed ahead of other potential risks—such as risking a political climate which deprioritizes personal liberties, such as freedom of movement—demonstrates the value commitments of these bodies.

Faced with such risks, sources noted the use of public health orders as a method of ensuring public safety—measures enacted at both the state/territory and federal levels that dictate under what conditions quarantine is instituted, how movement is curtailed, and under what conditions individuals can exercise free movement (Babones 2020a; Fotheringham et al. 2021; Senate Select Committee on COVID-19 2021; House of Representatives 2021; Pelkas 2010; Australian National Audit Office (ANAO) 2021; Carter 2020). The curtailing of these freedoms requires that the risks of harm are sufficiently great or widespread to be legally justified. To ensure compliance with these orders, public awareness of risks to health was commonly emphasized as a precondition, as only in cases where the risks are sufficiently high can the public be expected to cooperate (Davis, Stephenson, and Flowers 2011; Department of the Prime Minister and Cabinet 2019; Department of Health 2020; Department of Health and Ageing 2011; Moloney and Moloney 2020; Letts 2006).

Uncertainty was decoupled from the notion of risk in a minority of sources. In the case of a novel disease of pandemic potential, uncertainty refers to not only the *probabilities* of outcomes being unknown but so too the precise nature of those outcomes (Waller, Davis, and Stephenson 2016; Carney and Bennett 2013). This uncertainty, common to all emerging diseases, suggests parallels to the notion of “fog of war,” a term originally used to describe the uncertainty and confusion experienced by military planners during wartime (Waller, Davis, and Stephenson 2016, 101). At the time of the creation of the Australian Health Sector Emergency Response Plan for Novel Coronavirus in February 2020, many details were unknown, including the basic reproduction number, estimated at the time to be between 1.4 and 2.5 (Department of Health 2020). This meant that various assumptions needed to be made, and suggestions drawn up in response to likely scenarios. For example, if clinical severity proved to be like that of Spanish flu, widespread responses would be required, including emergency legislation to “support outbreak specific activities”; the document, however, does not specify the nature of these activities.

Risk mitigation plays a major part in decisions to control the movement of individuals who may have been exposed to, or exhibit symptoms of, a Listed Human Disease—that is, a disease listed under the *Biosecurity Act 2015* that is both communicable and likely to cause grave damage to human health (Ng and Gray 2021; Parliamentary Joint Committee on Human Rights 2020a, 2020b; Carter 2020; Walker 2020; Australian National Audit Office (ANAO) 2021). The decision to quarantine returning Australians from China on Christmas Island serves as a case in point (Holley, Coatsworth, and Lipman 2021; Milne et al. 2021; Gussen 2021; Van Nguyen et al. 2022). As noted by Holmes in the *ANZCA Bulletin* (2020), the use of Christmas Island to house these returning Australians entailed a risk of infection to both those returning travellers and staff when compared to alternatives, such as home detention—primarily because individuals were quarantined with other potentially infectious travellers in facilities not specifically designed for quarantine. Nonetheless, the decision was made to house these Australians offshore to mitigate risk to the broader community (Moloney and Moloney 2020; Holley, Coatsworth, and Lipman 2021; Dehm, Loughnan, and Steele 2021; Costantino, Heslop, and MacIntyre 2020; Stobart and Duckett 2021).

Discussions of risk that one may consider intuitively important—such as considering risks and uncertainty around gender-based violence in the wake of quarantine and other restrictions on movement—did not figure highly at all within the results our methodology generated. We speculate that the reason for this was likely our focus on how government and associated health bodies have wielded the concept of health security in framing and justifying their reactions to pandemics. We note that work has been done on this topic elsewhere (Mittal and Singh 2020; Pfitzner, Fitz-Gibbon, and True 2020; Rees and Wells 2020; Dlamini 2021; Pfitzner, Fitz-Gibbon, and True 2022).

To summarize, values guiding responses in the literature include the importance of minimizing risk to the public of significant harm, especially in cases of uncertainty; a deep concentration upon on risks to health, life, and the economy, including risks to international trade and domestic employment, ahead of other considerations of harm; the importance of risk mitigation in decisions to control the movement of individuals who may have been exposed to or exhibit symptoms of a Listed Human Disease; and a comparative lack of consideration of other risks, such as the risk of gender-based violence.

### Free Movement, Safety, and Security

Quarantine (here distinguished from isolation, as the latter can be understood to refer to segregating only the infected, while the former includes those who were, or could be, exposed) was standardly conceptualized as a necessary measure to prevent the spread of communicable disease by government and health authorities, as the alternative—allowing the unchecked spread of a communicable disease—would lead to a predictable “increase in collective morbidity and mortality” (Bennett 2009; Blakely et al. 2020; Guy and Hocking 2006; Chen 2018; Letts 2006, 131; Pelkas 2010; Moloney and Moloney 2020; Victorian Government Board of Inquiry into the COVID-19 Hotel Quarantine Program 2020; Czeisler et al. 2021; MacIntyre 2020a; Jefferies, McAdam, and Pillai 2021). This is not without cost, however, as quarantine and related measures limit freedom of movement (amongst other freedoms) as a means of ensuring the safety of the broader population (Department of Health 2020; Carter 2020; Parliamentary Joint Committee on Human Rights 2020a, 2020b; Dehm, Loughnan, and Steele 2021; Guy and Hocking 2006; Pelkas 2010; Department of the Prime Minister and Cabinet 2019; Dzankic and Piccoli 2020; Schwarz 2020; House of Representatives 2021).

Whatever measures are used to increase safety within a pandemic will likely impinge upon some freedoms (Parliamentary Joint Committee on Human Rights 2020a, 2020b; Letts 2006, 131). Sources noted that quarantine measures thus require the exercise of caution to ensure they do not impinge unduly and excessively upon freedoms and rights, an exercise in striking the right balance (Bennett 2009; Fotheringham et al. 2021; Davis, Stephenson, and Flowers 2011; Parliamentary Joint Committee on Human Rights 2020b; Guy and Hocking 2006; Parliamentary Joint Committee on Human Rights 2020a; Pelkas 2010; Gussen 2021; Dehm, Loughnan, and Steele 2021; Jefferies, McAdam, and Pillai 2021; Tomkins 2020; Higgins 2022). In terms of identified rights, these ranged from rights to life, health, and security (Legislative Assembly of the Northern Territory 2020; Parliamentary Joint Committee on Human Rights 2020a, 2020b; Dept. of the Prime Minister and Cabinet 2019; House of Representatives 2021; Babones 2020b; Andrews 2021; Victorian Government Board of Inquiry into the COVID-19 Hotel Quarantine Program 2020; Bowen 2020; Morrison 2020; Hunt and Kelly 2020; House of Representatives 2008b; Department of Health and Ageing 2006; Hunt and Murphy 2020; Kelly 2021; Legislative Assembly of Western Australia 2020a; Legislative Assembly of Western Australia 2020b); freedom and liberty, especially freedom of movement (Parliamentary Joint Committee on Human Rights 2020b; Jamrozik and Heriot 2020; Parliamentary Joint Committee on Human Rights 2020a; Pelkas 2010; Boucher et al. 2021; Hicks 2021; Lelliott, Schloenhardt, and Ioannou 2021; Bennett 2021); and economic rights (Bennett 2009; Boyd, Baker, and Wilson 2020; Bashford and Howard 2004; Ly, Selgelid, and Kerridge 2007; Department of the Prime Minister and Cabinet 2019; House of Representatives 2008a; MacIntyre 2020a; Bennett 2021). Some sources noted that Australia has no federal bill of rights, which means that there are no guaranteed procedural (or substantive) protections from incursions from the state (Bennett 2009; Guy and Hocking 2006; Hicks 2021; Babie and Russo 2020). As the powers exercisable at the federal level are largely discretionary and wide-ranging in their scope, sources identified a danger that incursions against individual rights will go largely unchallenged (Guy and Hocking 2006; Chen 2018; Pelkas 2010; Carter 2020; Dehm, Loughnan, and Steele 2021; Dzankic and Piccoli 2020; Schwarz 2020; Gray 2021; Murphy and Arban 2021).

Official bodies, as explored within academic studies, parliamentary speeches, and other official communiqués generally emphasized safety as being their primary concern, trumping freedom of movement and other freedoms ( Legislative Assembly of the Northern Territory 2020; Parliamentary Joint Committee on Human Rights 2020a, 2020b; Department of the Prime Minister and Cabinet 2019; House of Representatives 2021; Babones 2020b; Andrews 2021; Victorian Government Board of Inquiry into the COVID-19 Hotel Quarantine Program 2020; Bowen 2020; Morrison 2020; Hunt and Kelly 2020; House of Representatives 2008b; Department of Health and Ageing 2006; Hunt and Murphy 2020; Kelly 2021; Legislative Assembly of Western Australia 2020a; Legislative Assembly of Western Australia 2020b). For example, the Parliamentary Joint Committee on Human Rights analysed the human rights impact of COVID-19 measures and claimed that the right to life requires Australia to take positive measures in the protection of life and health (Parliamentary Joint Committee on Human Rights 2020a). The committee (and other sources) noted that these measures may also limit freedom of movement, alongside other freedoms, and that this decision is in line with international law, which stipulates that such freedoms are subject to permissible limitations if the measures are shown to be reasonable, necessary, and proportionate (Parliamentary Joint Committee on Human Rights 2020b; Bennett, Carney and Bailey 2012; Babones 2020b; Parliamentary Joint Committee on Human Rights 2020a; Lelliott, Schloenhardt, and Ioannou 2021; Bennett 2021; Murphy and Arban 2021). Their analysis of this balancing of freedom and safety offered the following:

The committee considers that legislation taken to control the entry, establishment or spread of COVID-19 is likely to promote and protect the rights to life and health. The right to life requires the State to take positive measures to protect life. The United Nations Human Rights Committee has stated that the duty to protect life implies that State parties should take appropriate measures to address the conditions in society that may give rise to direct threats to life, including life threatening diseases. (Parliamentary Joint Committee on Human Rights 2020a, 2)

This links to the related claim that quarantine can be understood as a guarantor of rights—that is, that without quarantine, and other restrictive measures, the rights to health and life cannot be protected during a pandemic (Department of the Prime Minister and Cabinet 2006; Parliamentary Joint Committee on Human Rights 2020b; 2020a; Lelliott, Schloenhardt, and Ioannou 2021). In the case of the rights analysis produced by the Joint Committee, this reasoning was used to justify decisions taken at the beginning of the pandemic, as the alternative was to risk the health and life of others by allowing the spread of an unknown disease (Parliamentary Joint Committee on Human Rights 2020b; 2020a).

Explicit and implicit values explored in the above can thus be summarized as a commitment to quarantine as a justified measure to prevent the spread of communicable disease; the identification of a range of rights impacted by such measures, some positively—the rights to life, health, and security—and others negatively (freedom of movement and economic rights); the primacy of safety as a trumping consideration ahead of other competing goods; and that (despite appearances) restrictive measures, such as quarantine, can be understood as a guarantor of rights.

### Legitimacy

For restrictive measures to be put into practice, they must be legitimated by reference to some justificatory framework. A justificatory framework provides an evaluative basis for determining the legitimacy of restrictions and helps to ensure that they are necessary and proportional to the threat that they are intended to address. One major source of legitimation for restrictive measures in response to public health risks we found was the blending of health considerations with *national* security by appealing to the notion of *health* security, highlighting the fact that disease outbreaks have the potential to destabilize national security just as surely as terrorism or military invasion (Babones 2020b; Brew and Burton 2004; Behm 2020; House of Representatives 2020a; Joint Standing Committee on Foreign Affairs, Defence and Trade 2020; Senate Select Committee on COVID-19 2020b). Sources noted that there could be an inherent tension between balancing national security and public health, insofar as the former focuses on maximizing top-down control and limiting information flow, while the latter relies upon openness and transparency through effective communication (Carter 2020; Chen 2018; Saul et al. 2020). More critical approaches argued that moving from an explicitly “health” framing to one akin to national security has potential drawbacks (Boyd, Baker, and Wilson 2020; Davis, Stephenson, and Flowers 2011; Carney and Bennett 2013; Pelkas 2010; Chen 2018; Brew and Burton 2004; Bennett, Carney and Bailey 2012).

In particular, one commonly noted issue is that this conflation of health and national security encourages a myopic focus upon measures that confer direct benefit to Australia, ahead of cooperative regional or international responses (Bashford 2003; Davis, Stephenson, and Flowers 2011; Waller, Davis, and Stephenson 2016; Pelkas 2010; Chen 2018; Brew and Burton 2004; Kamradt-Scott 2018). As Behm (2020) notes, the very notion of security encourages a kind of parochial focus on the nation state and its borders. Nonetheless, a commonly noted counterpoint was that at least part of the explicit motivation for adopting laws and opting into international legal instruments was for the purposes of protecting and defending narrow Australian interests for the benefit of the Australian public (Kamradt-Scott 2018; Australian Government 2006; Schierhout et al. 2017). For example, in their explanatory memorandum to the World Health Organization’s International Health Regulations (IHR), the Federal Government claimed

The IHRs […] outline a framework for identifying public health risks and events of international concern, and for implementing a coordinated public health response to them. Through this framework, Australia will gain access to important information and assistance relating to public health risks. Australia would also be in a strong position to contribute to the formulation and implementation of a coordinated and integrated international response. (Australian Government 2006, para. 7)

Where policy decisions take place in a democratic country, public acceptance is a key determinant of the legitimacy of any policy decision. Public compliance and acceptance were both picked out as key features, with the former resulting from the latter (Davis, Stephenson, and Flowers 2011; Department of Health 2020; Belinda Bennett, Carney and Bailey 2012; Ly, Selgelid, and Kerridge 2007; Department of Health and Ageing 2006; Department of the Prime Minister and Cabinet 2019; Halton et al. 2021; Letts 2006; Carney and Bennett 2013; Australian National Audit Office (ANAO) 2021; Department of Health and Ageing 2011). In terms of gaining acceptance, a common claim was that the public will likely accept any necessary measures that minimize the impact or spread of disease (Davis, Stephenson, and Flowers 2011; Bennett, Carney, and Bailey 2012; Department of Health 2020). Gaining public support for pandemic measures is thus dependent upon whether Australian citizens can reasonably expect to benefit—that is, whether restrictive measures will succeed, or are likely to succeed, in slowing or stopping the spread of communicable disease (Bennett, Carney and Bailey 2012; Guy and Hocking 2006; Joint Standing Committee on Foreign Affairs, Defence and Trade 2020; Babones 2020b; Jamrozik and Heriot 2020; Parliamentary Joint Committee on Human Rights 2020a, 2020b; Department of Health and Ageing 2011; Pelkas 2010; Hicks 2021; Gray 2021).

The values underpinning the above can be summarized as, first, that a major (contested) source of legitimation for restrictive measures is the notion that health security is usefully contrasted with *national* security; second, that this move can be utilized to motivate public acceptance of measures that would otherwise garner little support; third, that this public support is necessary if measures will be accepted (or, indeed, acted upon).

## Discussion

The values underlying various interventions proposed by government bodies, political parties, and health services requires re-examination and, crucially, explicitly *ethical* scrutiny (Jamrozik and Heriot 2020; Pelkas 2010; Lelliott, Schloenhardt, and Ioannou 2021). As values work to shape the range of acceptable arguments and positions within a problem space, one of the tasks of ethics is to seek out these operant values and subject them to critical analysis, in recognition of the fact that implicit values exert pressure on decisions in ways that are not immediately obvious (Brownstein 2019). In a democracy that aspires to empower the populace, the underlying reasons for decisions are as important as the decisions themselves. Based on the results of our review, we picked out three underlying areas that we take to be particularly worthy of additional scrutiny: first, how harm is understood within this literature; second, how safety and security are conceptualized; finally, how rights analyses have been conducted. We picked these three areas due to their recurring nature and because of how central these claims are in helping shape Australia’s response to COVID-19.

### Accurate Portrayal of Harms

That some public health intervention causes harm is not sufficient reason to ethically invalidate it, but it does mean that the intervention requires careful ethical justification (Austin 1971). Harm can be understood in a variety of ways, and which understanding is utilized will shape the range of acceptable actions open to decision-makers.

Harm could be understood narrowly in terms of physical outcomes—that is, a physicalist understanding of harm—a minority position within moral philosophy (Mattiasson and Andersson 1995; Schein and Gray 2018). Most obviously, falling sick from a preventable and dangerous disease would count as a physical harm. It may seem, then, that interpreting the harm of COVID-19 interventions in the name of health security involves answering a reasonably simple question: whether the physical harms of unchecked transmission are sufficiently grave to warrant controlling movement, association, and other related liberties. If this was the best, or only, concept of harm available, then it would seem as if there were little further work to be done.

However, consider two further renderings of harm that are popular within the ethics literature. First, there is the consequentialist notion that harm is best understood as a failing to satisfy a preference (Hare 1981; Sinnott-Armstrong 2019). Under this understanding, individuals are harmed when their preferences are unjustifiably overridden. This wider conception of harm encompasses harms to one’s physical person but takes the avoidance of physical injury as simply another preference amongst many. We also have preferences regarding our liberties, and interference with these preferences amounts to harm that requires justification. Another equally well-represented view of harm within the ethics literature centres upon the setting back of another’s interests (Feinberg 1990). Under this view, we harm another when we set back their goals or projects, such that they are left worse off after our intervention. Amongst these “interests” we find, again, physical safety and health, counted among other interests, such as interests in liberty, association, and so on.

This lack of precision within governmental and health literature as to how harms are understood and weighted against predicted benefits means that there is an inherent difficulty in evaluating the ethical plausibility of the reasoning behind measures chosen by government and associated agencies. This point is exacerbated by the fact that distinct renderings of harm offer up distinct answers as to how to perform this weighting of harms and benefits. While a physicalist would simply weigh harms to health or life against temporary restrictions, a preference satisfaction theorist could count the number and strength of the preferences under consideration and act to ensure the majority have their preferences met. In turn, under most interest-based views it is considerably more difficult to justifiably weigh the interests of distinct individuals against each other, especially when these interests are equally fundamental (Williams 1973). Dworkin (1993) argues that not all interests are created equal: some interests are morally more significant. According to Dworkin, “critical” interests are those that undergird living a good life on the whole, which are to be offered special protections from outside interference (Dworkin 1993, 201–202). Returning to the issues of health security and COVID-19, perhaps one could argue that liberty is best understood alongside other critical interests, such as safety, and we cannot justify imposing restrictions on liberty simply for the good of the population at large, just as we cannot demand that individuals give up their critical interests for the sake of the critical interests of others.

The point here is not that an interest-based account is the right one, nor that other alternative accounts of harm are meritless. Instead, the claim is that any restriction placed upon the public in the name of health security requires careful work to unpack the harms that will be produced by those measures, such that others can reasonably agree or object to both the characterization and balancing of those harms. As noted above, discussions of harm from governmental and health bodies largely focused upon a limited view of harm, with particular focus placed upon economic harms and harms likely to arise from risks to health and life. As noted directly above, alongside this finding, there is the related issue that how we define harm will necessarily affect how potentially harmful actions are categorized and the weightiness of considerations that are required to justify those harms. In leaving the definition of harm implicit, these Australian bodies fail to provide the information needed to invite thoroughgoing ethical analysis. Our impression of the governmental literature is that it implicitly and artificially defines harms in a physicalist manner, thereby overlooking alternate interpretations of harm that seem (to us at least) no less pressing. Without this prior work of making the implicit explicit, discussion of “harm” remains nebulous and contestable in policy and practice.

### Safety and Security

The surveyed literature shares some commonalities with other well-known approaches to safety and security. Safety and security are often understood in the medical or public health contexts as the absence of hazards, where a hazard refers to a state of affairs that could bring about some morally significant harm, such as loss of flourishing or loss of life (Runciman, Merry, and Walton 2007). Safety/security is thus conceptualized as the end result of actions to mitigate or eliminate unacceptable risk (Fischhoff, Watson, and Hope 1984; Slovic 1999).

In the surveyed literature outlined above, safety and security were often regarded as outweighing other goods in the face of a pandemic, even if these interventions restrict important freedoms or rights. Despite this common narrative, it is worth emphasizing that this weighting of security and safety is a contentious value judgement (Waldron 2006; Jennings 2009; Coggon 2021). Safety necessarily involves minimizing or eliminating specifically *unacceptable* risks, as we cannot hope to eliminate all risks whatsoever. In determining what is and is not an acceptable or unacceptable sacrifice in favour of safety we must appeal to a value judgement that some goods are sufficiently weighty to justify this sacrifice or that the likely harms simply outweigh the benefits. There is no value-free fact of the matter that safety does, or should, trump other considerations, and yet this appears to be a common assumption.

One might respond that safety is a precondition for the exercise of other rights and freedoms (Leaning and Arie 2001; Crawford 2014). As noted above, some rights or interests appear more basic than others. One could point to something akin to Maslow’s hierarchy of human needs, which interprets safety and security as more fundamental than other needs, including freedom (Maslow 1943). Similarly, Nussbaum and Sen argue that safety, amongst other basic capabilities, are preconditions for well-being (Sen 1993; Nussbaum 2000). So, if restrictive measures such as quarantine are the only practicable way to ensure a right to life and security, then these measures inherit the moral significance of those goods they bring about. It remains to be seen, however, whether this kind of hierarchy is ethically plausible, and reasonable persons can disagree both as to the appropriateness of a hierarchy as well as whether liberty should be considered as basic as other goods (c.f. Wahba and Bridwell 1976).

Alternatively, one might appeal to the broad base of acceptance of these restrictive measures within the population. Multiple polls throughout the pandemic demonstrated that border closures, amongst other restrictive measures, enjoyed public support in Australia during the pandemic (Hicks 2021; Kassam 2021; The Australia Institute 2020). However, as Hicks (2021) notes, popular support can mean that governments are perversely incentivized to adopt popular, but ethically suspect, measures.

This appeal to safety as outweighing other goods should thus be explicitly discussed as a value judgement, rather than treated as a foregone conclusion. Defending safety as trumping other interests or goods, in other words, requires that decision-makers provide public *arguments* that safety outweighs other considerations. An actual defence of these measures would likely require careful demonstration that while liberty is an important right, it is outweighed by considerations of safety, and for particular, well-founded, reasons. Some sources noted that we are far from this ideal, as there are no clear pathways to appeal a decision to quarantine or coerce an individual, and states have proven unwilling to publicize restrictive health measures or offer accountability mechanisms (Guy and Hocking 2006; Jamrozik and Heriot 2020; Carter 2020; Pelkas 2010; Hicks 2021). For example, Carter found that many Australian jurisdictions are unwilling to release data regarding the use and targets of public health control orders, with South Australia going so far as to foreclose the possibility of ever releasing such data (Carter 2020). A public defence of this weighting of safety ahead of other considerations requires careful finessing of arguments and subjecting this reasoning to public debate and scrutiny (Bennett 2009; Carney and Bennett 2013; Pelkas 2010; Carter 2020; Dehm, Loughnan, and Steele 2021; Hicks 2021). This requires a commitment to public transparency and accountability.

### Articulating the Values Underpinning the Balancing of Rights

As noted above, various political and health organizations have engaged in an implicit analysis of conflicting rights. Measures that restrict freedoms—freedoms of movement, of association, of choice, and so on—necessarily impinge upon rights (Cameron et al. 2021). Any decision in response to a public health emergency will therefore require the balancing of rights against each other, with an eye towards an equitable balancing of competing considerations. Quarantine is no different and this explains why conversations of rights and freedoms figure highly in the surveyed literature, especially those that are interested in motivating and justifying these measures (Department of Health 2020; Parliamentary Joint Committee on Human Rights 2020b; Guy and Hocking 2006; Department of the Prime Minister and Cabinet 2019; Parliamentary Joint Committee on Human Rights 2020a; House of Representatives 2021; Pelkas 2010; Carter 2020; Dehm, Loughnan, and Steele 2021; Dzankic and Piccoli 2020; Schwarz 2020; Babie and Russo 2020; Murphy and Arban 2021).

Take as our motivating example the designation of Christmas Island as a “human health response zone” and how this decision was made and justified to the public (Babones 2020a; Parliamentary Joint Committee on Human Rights 2020b; Hunt and Murphy 2020; Morrison 2020; Hunt and Kelly 2020; Parliamentary Joint Committee on Human Rights 2020a; Holmes 2020; Dehm, Loughnan, and Steele 2021; Senate Select Committee on COVID-19 2020b; Keneally 2020; Senate Community Affairs Legislation Committee, 2020; Murphy and Arban 2021). Primarily Australian Chinese citizens were housed in that facility for fourteen days, regardless of testing results, before being allowed to enter Australia proper. This plan was put into action on February 3, when around 240 Australians were evacuated to the facility alongside forty-three permanent residents, at a time when COVID-19 had already reached the Australian mainland, with twelve identified cases (Shelton and Birtles 2020). Similarly, in May 2021, the Federal Government announced a ban on returning travellers, including Australian citizens, from India in response to the emerging Delta variant (Halton et al. 2021; Stobart and Duckett 2021; Higgins 2022; Rizzi and Tulich 2021; Jefferies, McAdam, and Pillai 2021). This decision, much like the decision in February 2020, sparked debate regarding the transparency of government decision-making, as well as the motives lying beneath these decisions (Higgins 2022; Rizzi and Tulich 2021; Gunia 2021; Jefferies, McAdam, and Pillai 2021).

One might conclude that the decision to treat returning travellers—the majority of whom were Chinese Australians or Indian Australians—can be readily explained in terms of balancing rights. On the one hand, we find freedom of movement and association, and on the other we find the right to live free from communicable, dangerous disease. This position may explain why some sources (as noted above) claimed that quarantine is a guarantor of rights. The problem with this response is that it is unclear why the rights are balanced in this manner, given that reasonable people disagree as to their weightings, as well as their applicability.

We return to this example insofar as it demonstrates how values underlying decisions can be put into action. The dialectic surrounding Christmas Island is emblematic of a general tendency to claim, in passing, that quarantine and other restrictive measures need to be balanced against human rights without explaining, or justifying, how this balancing is achieved. The problem, as we see it, is that any thoroughgoing analysis—especially one that targets actual legislative instruments—cannot simply *claim* that rights are being weighed appropriately. Instead, this balancing requires demonstration of how an outcome is permissible by reference to a particular underlying (likely, ethical) theory. This is necessary, and useful, insofar as it effectively demonstrates the reasoning of those performing the analysis and opens these bodies up to reasonable—and informed—critique. In the case of Christmas Island, no such demonstration was forthcoming, leading to considerable consternation from these returning travellers as to why they were treated differently to other returning Australians (Xiao 2020; *BBC* *News* 2020). Determining the acceptability of these restrictive measures is important, given their scope, as well as their possible conflict with the International Covenant on Civil and Political Rights that no individual shall be arbitrarily denied the right to return to their country (United Nations General Assembly 1966). The deployment of these powers can have, and has had, enormous impact upon those subjected to them, and yet decision-makers are granted considerable leeway in their decisions with little public oversight (Guy and Hocking 2006; Chen 2018; Carter 2020; Dehm, Loughnan, and Steele 2021; Victorian Government Board of Inquiry into the COVID-19 Hotel Quarantine Program 2020; Pelkas 2010; Dzankic and Piccoli 2020; Schwarz 2020; Gray 2021).

If the exercise of these powers is the result of careful balancing of rights, then these rights are being balanced outside of the public eye—a situation we take to be inherently open to abuse. There is a strong *prima facie* case that if these powers are to be granted to governments or health bodies, then they can, and should, be subject to public scrutiny, as this is necessary for a full and frank discussion of how these rights *ought* to be balanced, both according to ethical precepts and in the light of international law.

This relates to a broader value commitment within the literature that treated the concept of health security as a sufficient justification. Successive Australian governments have appealed to health security, as a natural extension of national security, to justify the expansion and exercise of considerable power (Davis, Stephenson, and Flowers 2011; Chen 2018; Brew and Burton 2004; Babones 2020b; Carter 2020; Department of Health and Ageing 2011; Australian National Audit Office (ANAO) 2021; Bennett 2021). This prioritization of health security seems to override other considerations, including individual liberty and rights. There are many critiques possible here, such as noting that the increasing “securitization” of health leads to negative outcomes, both ethically and prudentially (Brew and Burton 2004; Davis, Stephenson, and Flowers 2011; Waller, Davis, and Stephenson 2016; Kamradt-Scott 2018). More pointedly, appealing to health security merely pushes the question back a step, as work remains to explain why health security *should* win out against alternative arguments.

Our motivating example serves as a case in point. To maintain health security these travellers were placed onto Christmas Island where they ran a risk of infection from other travellers, given that there was no way to keep families entirely separated in the facilities (Holmes 2020). This, coupled with the fact that Christmas Island lacks a hospital capable of taking care of COVID-19 patients (Doherty 2020), means that a very strong argument indeed would need to be provided to justify this decision, given the risks foisted upon these travellers to benefit others. It is not enough, as is sometimes supposed, to point out that the risks of travelling to Christmas Island were comparably minor to remaining in Wuhan (MacIntyre 2020b), as another option remained: home quarantine. So, a thoroughgoing description of this decision would need to explain why it was right to prioritize the safety of the mainland ahead of the safety (and rights) of those who were subjected to quarantine. Until this necessary deliberation is done publicly, it is difficult—if not impossible—to determine whether these actions are defensible as they occurred. Indeed, our overall position here is not to claim that it would be impossible to justify a quarantine system similar to that undertaken by the Australian Government in the wake of COVID-19. Our claim is altogether more modest: that the articulation, and defending, of *how* rights were balanced (as well as the particular *weighting* of these rights in comparison to one another) is a vital part of a defensible democratic system. A similar point has been made elsewhere in the context of medical resource allocation (Gruskin and Daniels 2008; Badano 2014; Pratt and Hyder 2017). There the claim is that resource allocation decisions, such as to prioritize certain forms of healthcare or particular populations, invariably impinge upon the rights of others who would otherwise benefit from these resources. As a result, publicity is required regarding both the final decision made by official bodies, as well as the rationale behind those decisions, allowing for improvement over time in the wake of concerted scrutiny (Gruskin and Daniels 2008). The lesson is no less pressing within this context.

## Conclusion

Our critical interpretive review identified three primary themes: the centrality of risks; how safety is conceptualized; and what concepts are taken as legitimating by governments and associated bodies. In our discussion, we highlighted the way that normative concepts, such as “health security,” are marshalled to conceptualize the nature of pandemics and the Australian Government’s response to them. This allowed us to offer key ethical critiques of the dominant narratives that emerged. As we showed, harms were often left nebulous, safety was emphasized—but not publicly defended—at the expense of competing values, and rights analysis often failed to adequately demonstrate and defend how rights were compared and contrasted. Elucidating, defending, and exploring these spheres of value and how they are employed in public decision-making in the name of health security will only serve to encourage more informed, and ethically defensible, policy implementation.
